# Protective Association

**Published:** 1884-09

**Authors:** 


### PROTECTIVE ASSOCIATION.

It would seem as though dentists should have little to fear from patents obtained by perjury and fraud. But it has so often been the case that men who have used a device for perhaps a score of years, have suddenly found themselves confronted by an agent armed with a patent, and have been forced to pay a royalty upon their own inventions, that it is perhaps the best policy to resist such impositions at their outset. Patent lawyers are cunning and unscrupulous, and the Goodyear Dental Vulcanite Company taught us that when, by any means, collusive or otherwise, a few decisions in circuit courts have been obtained, it is hopeless to expect that new trials will be decided upon their merits. Every decision gained is a precedent established. The Richmond patents on tooth crown and bridge work now threaten us, and a protective association has been formed to fight them, of which Dr. W. H. Dwinelle is president, and Dr. J. M. Crowell, secretary. Dentists desiring further information may address either, at New York.

